# Carbapenems and colistin resistance genes isolated in *Musca domestica* from a garbage dump near a hospital in Lima

**DOI:** 10.17843/rpmesp.2024.412.13257

**Published:** 2024-06-12

**Authors:** Miguel A. Alarcón-Calle, Víctor L. Osorio-Guevara, Ramsés Salas-Asencios, José Yareta, Pool Marcos-Carbajal, María E. Rodrigo-Rojas

**Affiliations:** 1 Biology Research Laboratory, Faculty of Natural Sciences and Mathematics, Universidad Nacional Federico Villarreal, Lima, Peru. Universidad Nacional Federico Villarreal Biology Research Laboratory Faculty of Natural Sciences and Mathematics Universidad Nacional Federico Villarreal Lima Peru; 2 Biotechnology Laboratory, Faculty of Natural Sciences and Mathematics, Universidad Nacional Federico Villarreal, Lima, Peru. Universidad Nacional Federico Villarreal Biotechnology Laboratory Faculty of Natural Sciences and Mathematics Universidad Nacional Federico Villarreal Lima Peru; 3 Laboratory of Molecular Biology Research, Professional School of Medicine, Universidad Peruana Unión, Lima, Peru. Universidad Peruana Unión ">Laboratory of Molecular Biology Research Professional School of Medicine Universidad Peruana Unión Lima Peru; 4 Laboratory of Molecular Epidemiology and Genomics, Institute for Research in Biomedical Sciences, Faculty of Human Medicine, Universidad Ricardo Palma, Lima, Peru. Universidad Ricardo Palma Laboratory of Molecular Epidemiology and Genomics, Institute for Research in Biomedical Sciences Faculty of Human Medicine Universidad Ricardo Palma Lima Peru

**Keywords:** Houseflies, Drug Resistance, Colistin, Carbapenem

## Abstract

The objective was to determine the presence of carbapenem resistance genes and plasmid resistance to colistin (mcr-1) in bacteria isolated from *Musca domestica* in a garbage dump near a hospital in Lima, Peru. Bacteria with phenotypic resistance to carbapenemics were isolated on CHROMagar mSuperCARBATM medium and colistin resistance profiling was performed using the colistin disk elution method. Detection of *bla*KPC, *bla*NDM, *bla*IMP, *bla*OXA-48, *bla*VIM and mcr-1 genes was performed by conventional PCR. The antimicrobial susceptibility profile was determined using the automated MicroScan system. We found that 31/38 strains had phenotypic resistance to carbapenemics and 26/38 strains had phenotypic resistance to colistin with a minimum inhibitory concentration ≥ 4 µg/ml. Finally, we identified seven bacterial strains with carbapenem resistance genes (OXA-48 and KPC) and one bacterial strain with plasmid resistance to colistin (mcr-1). One Escherichia coli strain had three resistance genes: KPC, OXA-48 and mcr-1.

## INTRODUCTION

The World Health Organization (WHO) warns about antimicrobial resistance (AMR) globally, due to the increase of multidrug resistant (MDR) bacteria and the decrease of therapeutic options. This causes approximately 700,000 deaths annually, with an estimated 10 million deaths per year by 2050, affecting socioeconomic and demographic factors ^1^.

Carbapenems have been considered as “the last and most effective resource to treat bacterial infections” ^2^; however, the number of carbapenemase-producing bacteria, such as *Klebsiella pneumoniae*, *Acinetobacter baumannii* and *Pseudomonas aeruginosa*, has increased ^3^. Several enzymes, such as Verona metallo-beta-lactamase (VIM), *Klebsiella pneumoniae* carbapenemase (KPC), Imipenemase (IMP), Oxacillinase (OXA-48), and New Delhi metallo-betalactamase (NDM), have been described since the 1990s with global dissemination, including Peru ^4^. The use of colistin as a last therapeutic resort has been hampered by the emergence of the resistance gene Mobile Colistin Resistance (mcr-1) in China in 2015, spreading through different origins ^5^. The first report in Peru related to the mcr-1 gene was made in *Escherichia coli* (*E. coli*) isolated from a urine culture sample ^6^.

Houseflies are mechanical and biological vectors of bacteria, carrying up to 500,000 bacterial agents, including clinical pathogens ^7^. Zhang *et al*. detected colistin resistance genes (mcr-1 to mcr-3) in flies, with 34.1% of bacteria positive for mcr-1 ^8^.

In Brazil, the *bla*NDM-1 gene was reported for the first time in *Musca domestica* in *E. coli* from an urban center in Rio de Janeiro ^9^. However, there are few reports on carbapenemase-producing and colistin-resistant bacteria carried by *Musca domestica*. There are no studies in Peru on vectors as a source of dissemination of resistance genes to this group of antibiotics. This study seeks to identify carbapenemase-producing genes and mcr-1 plasmid gene in bacteria isolated from flies from a garbage dump near a hospital in Lima, Peru.

**KEY MESSAGES**

Motivation for the study. The presence of antibiotic resistance genes in bacteria isolated from common flies is a potential public health hazard because it facilitates the presence and spread of antibiotic resistance genes in the environment.

Main findings. Thirty-eight bacterial strains identified in 14 species were isolated from within the fly bodies, of which 31 strains showed resistance to carbapenems and 26 strains showed resistance to colistin. Seven bacterial strains showed carbapenem resistance genes and one *Escherichia coli* strain had resistance to KPC, OXA-48 and mcr-1.

Implications. This is the first report of antibiotic resistance genes in bacteria carried by common flies in Peru.

## THE STUDY

The assays of this study were conducted at the Biology Research Laboratories of the Universidad Nacional Federico Villarreal and the Laboratory of Molecular Biology Research (LIBM) of the Universidad Peruana Unión during March to August 2023.

### Design and sample

We carried out an observational, cross-sectional, descriptive study, in which 30 houseflies collected in April 2023 from a garbage dump near the EsSalud Level II Hospital in Ate Vitarte, Lima, Peru (coordinates: -12.026535/-76.924063) were analyzed.

### House fly collection and identification

For the collection and identification of the flies, we used sticky plates with chicken as bait. Each fly was individually placed in 2-ml Eppendorf tubes and kept at 0 °C for 20 min to numb the flies and facilitate handling ^9^. They were then identified using Robert Moon’s taxonomic key ^10^.

### Culture, bacterial identification and susceptibility profiling

Flies were washed with sterile 1X PBS to eliminate external bacteria. The flies were then triturated and the homogenate was resuspended in PBS and centrifuged for 10 minutes. Them, 1000 µl of the supernatant was removed and placed in tubes with 3 ml of trypticase soy broth and incubated at 37 °C for 18 to 24 hours ^8^; 10 µl of broth was then removed with a calibrated loop and seeded on MacConkey agar plates. Bacterial identification was carried out by conventional biochemical tests: TSI, LIA, SIM, Citrate and Urea Agar.

Antibiotic susceptibility profiling and species confirmation, for strains with resistance genes determined by PCR, was performed with the automated Microscan® (AutoScan-4) system following manufacturing instructions. Eighteen antimicrobials were included in the susceptibility panel: amikacin (AK), gentamicin (GEN), tobramycin (TOB), cefepime (FEP), cefuroxime (CFX), ceftazidime (CAZ), cefotaxime (FOX), ampicillin (AMP), ampicillin with sulbactam (AMP/SUL), amoxicillin with clavulanic acid (AMC), piperacillin with tazobactam (PIP/TZ), imipenem (IMI), meropenem (MEM), ertapenem (ETP), aztreonam (ATM), ciprofloxacin (CIP), levofloxacin (LEV), trimethoprim/sulfamethoxazole (SXT) and colistin (COL). The results were interpreted according to Clinical and Laboratory Standards Institute (CLSI) guidelines ^11^.

### Phenotypic detection of carbapenemases and colistin resistance

Isolation of bacteria with phenotypic resistance to carbapenems was carried out on CHROMagar^TM^ mSuperCARBA^TM^ medium. To evaluate phenotypic resistance to colistin, we used 10 µg colistin discs in Müller Hinton broth with cations, following the Clinical and Laboratory Standards Institute (CLSI) 2023 guidelines, considering resistance with a cut-off point ≥ 4 µg/ml ^11^.

### Detection of *bla*KPC, *bla*NDM, *bla*IMP, *bla*OXA-48, *bla*VIM and mcr-1 genes

Finally, DNA was extracted using the immuPREP Bacteria DNA Kit (Analitikjena, Germany) and *bla*KPC ^12^, *bla*NDM ^13^, *bla*IMP ^14^, *bla*OXA-48 ^15^, *bla*VIM ^16^ and mcr-1 ^17^^)^ resistance genes were detected by Polymerase Chain Reaction in a T100™ thermal cycler (Biorad, USA), following standardized protocols in the laboratory (LIBM); in the case of the mcr-1 gene, the Iglesias protocol was used ^17^. The primers used for this process are detailed in Table 1. Multidrug-resistant bacterial strains were used as positive controls, such as *Klebsiella pneumoniae* derived from ATCC BAA-1705 (Microbiologics) for the KPC gene, *Escherichia coli* derived from ATCC BAA-2469 (Microbiologics) for the NDM gene. We also used strains provided by the National Reference Laboratory (*Laboratorio Intrahospitalaria*-LIH) of the National Institute of Health for each gene of interest (IMP, OXA-48, VIM and mcr-1) identified by PCR; and two negative controls: molecular grade water and *Escherichia coli* ATCC 25922 (Microbiologics).

**Table 1:** Primers used in this study.

Gene	Primer	Sequence 5’-3’	Amplicon	Reference
KPC	F	AACAAGGAATATCGTTGATG	916 bp	^12^
	R	AGATGATTTTCAGAGCCCTTA		
NDM	F	AGCACACTTCCTATCTCGAC	512 bp	^13^
	R	GGCGTAGTGCTCAGTGTC		
IMP	F	GGYGTTTWTGTTCATACWTCKTTYGA	404 bp	^14^
	R	GGYARCCAAACCACTASGTTATCT		
VIM	F	AGTGGTGAGTATCCGACAG	261 bp	^15^
	R	ATGAAAGTGCGTGGAGAC		
OXA-48	F	ATGCGTGTATTAGCCTTATCGG	438 bp	^16^
	R	GCGTGGTTAAGGATGAACAC		
MCR-1	F	CGGTCAGTCCGTTTGTTC	309 bp	^5^
	R	CTTGGTCGGTCTGTAGGG		

F: Forward, R: Reverse, KPC: *Klebsiella pneumoniae* Carbapenemase, NDM: New Delhi metallo-β-lactamase, IMP: Imipenemase, VIM: Verona integron-encoded metallo-beta-lactamase, OXA 48: Oxacillinase type 48 and MCR: Mobile Colistin Resistance.

### Electrophoresis

Amplification products were analyzed by 1.5% agarose gel electrophoresis (Cleaver Scientific) with SYBR Safe DNA Gel Stain as gel buffer, and 6X DNA Loading Dye as loading buffer. A 100 bp DNA(Trans) marker was used to assess DNA size, with a run at 100 V for 40 min.

### Statistical analysis

IBM SPSS Statistics 25.0 software was used to present frequency measures as percentages. Tables and figures were constructed using Microsoft Excel 2019.

### Ethical considerations

Approval by an institutional ethics committee was not required since no patient data were used.

## FINDINGS

We identified 14 species from the 38 bacterial strains that were isolated, as detailed in Table 2. *Klebsiella oxytoca* was the most prevalent species (10/38, 23.7%) followed by *Escherichia coli* (7/38, 21.1%).

**Table 2:** Frequency of isolated bacteria, phenotypic and genotypic detection related to carbapenemase production and plasmid resistance to colistin.

Bacteria	Phenotype			Genotype					
	Frequency (%)	mSuperCARBA ^TM^ (%)	Elution of colistin discs ≥4µg/ml (%)	Gene					
				***bla*KPC**	***bla*NDM**	***bla*OXA-48**	***bla*VIM**	***bla*IMP**	mcr-1
*Klebsiella oxytoca*	10 (23.7)	10 (32.3)	5 (19.2)	ND	ND	1	ND	ND	ND
*Escherichia coli*	7 (21.1)	7 (22.6)	5 (19.2)	1	ND	2	ND	ND	1
*Enterobacter aerogenes*	3 (7.9)	2 (6.5)	3 (11.5)	1	ND	ND	ND	ND	ND
*Citrobacter freundii*	3 (7.9)	2 (6.5)	2 (7.7)	ND	ND	ND	ND	ND	ND
*Enterobacter cloacae*	2 (5.3)	2 (6.5)	2 (7.7)	ND	ND	1	ND	ND	ND
*Klebsiella pneumoniae*	2 (5.3)	2 (6.5)	1 (3.8)	ND	ND	ND	ND	ND	ND
*Proteus vulgaris*	2 (5.3)	1 (3.2)	1 (3.8)	ND	ND	1	ND	ND	ND
*Pseudomonas aeruginosa*	2 (5.3)	1 (3.2)	1 (3.8)	ND	ND	ND	ND	ND	ND
*Pseudomonas fluorescens*	2 (5.3)	1 (3.2)	1 (3.8)	ND	ND	ND	ND	ND	ND
*Acinetobacter baumannii*	1 (2.6)	0 (0.0)	1 (3.8)	ND	ND	ND	ND	ND	ND
*Morganella morganni*	1 (2.6)	1 (3.2)	1 (3.8)	ND	ND	ND	ND	ND	ND
*Proteus mirabilis*	1 (2.6)	1 (3.2)	1 (3.8)	1	ND	ND	ND	ND	ND
*Proteus penneri*	1 (2.6)	0 (0.0)	1 (3.8)	ND	ND	ND	ND	ND	ND
*Serratia marcescens*	1 (2.6)	1 (3.2)	1 (3.8)	ND	ND	ND	ND	ND	ND
Total	38 (100.0)	31 (100.0)	26(100.0)	3	0	5	0	0	1

ND: Not Detected, KPC: *Klebsiella pneumoniae* Carbapenemase, NDM: New Delhi metallo-β-lactamase, IMP: Imipenemase, VIM: Verona integron-encoded metallo-beta-lactamase, OXA 48: Oxacillinase type 48 and MCR: Mobile Colistin Resistance.

Thirty-one strains of bacteria with phenotypic resistance to carbapenems were isolated: *Klebsiella oxytoca* (10/31, 32.3%) and *Escherichia coli* (7/31, 22.6%) were the most prevalent. In addition, 26 strains showed phenotypic resistance to colistin; where *Escherichia coli* and *Klebsiella oxytoca* showed the highest resistance (5/26, 19.2%), all with cut-off point ≥4µg/ml.

**Figure 1:**
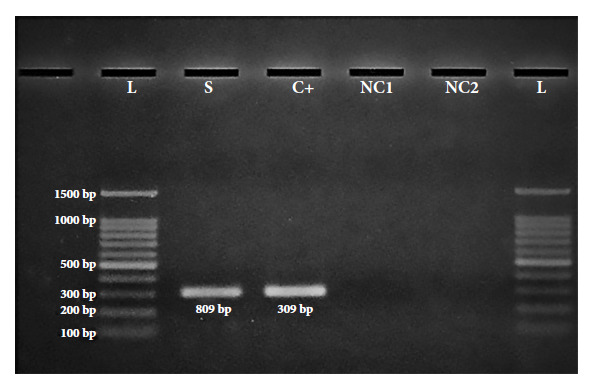
Electrophoresis results for the detection of the mcr-1 gene.

**Figure 2:**
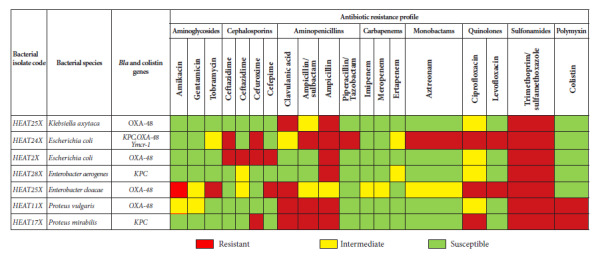
Genotypic and phenotypic resistance profile of carbapenemase-producing bacteria.

Seven bacterial strains with carbapenem resistance genes (OXA-48 and KPC) isolated on CHROMagarTM mSuperCARBATM were detected, of which the *bla*KPC gene was found in *Enterobacter aerogenes* (1/2) and *Proteus mirabilis* (1/1) and the *bla*OXA-48 gene in *Escherichia coli* (1/7), *Klebsiella oxytoca* (1/10), *Enterobacter cloacae* (1/2) and *Proteus vulgaris* (1/2). In addition, one *Escherichia coli* strain (1/7) had the resistance genes *bla*KPC, *bla*OXA-48 and the colistin-associated plasmid gene mcr-1, which would represent 3.8% of 1/26 strains. Details of the mcr-1 gene are presented in Figure 1 (electrophoretic run) and Figure 2 (heat map, phenotypic and genotypic resistance profile).

Regarding the antibiotic resistance profile, all isolates with detected resistance genes were resistant to 1 or 2 groups of antibiotics, as detailed in Figure 2. On the other hand, bacterial resistance to trimethoprim/sulfamethoxazole was found on 100% of the samples, 85.7% were resistant to ampicillin and 42.9% to ciprofloxacin, while there was sensitivity and/or intermediate resistance to ertapenem, imipenem and meropenem in all seven strains. Resistance to colistin was also observed in *Proteus* strains; however, this resistance is natural to the bacteria.

## DISCUSSION

Our results show that flies not only serve as dispersal vectors for different bacterial species, but also for bacteria carrying carbapenem-resistance genes to carbapenems and colistin (mcr-1).

In 2021, a study was conducted in Sudan, Africa on 300 flies collected from slaughterhouses and hospitals, and reported that, after examining the external and internal bacteria of each fly, 283 bacteria were identified in hospital flies and 366 bacteria in slaughterhouse flies; *E. coli* being the bacterium with the highest frequency of isolation ^18^. Despite the difference in the size of the bacterial sample isolated in our study, we detected 32.2% of *Klebsiella oxytoca* and 22.6% of *E. coli*, all carbapenemase-producing; therefore, they are the bacterial species most frequently carried by *Musca domestica*.

In 2004 and 2005, 780 flies were collected in markets and garbage dumps in Lima and Callao (Peru), and *Salmonella typhi*, *Shigella flexneri*, *Yersinia* enterocolitica and enteropathogenic *E. coli* were identified ^19^. These results allowed the authors to point out the importance of flies as mechanical vectors for the spread of bacteria in the environment. Our results also show *E. coli* as a bacterial isolate of *Musca domestica* that inhabit garbage dumps and that could also disperse resistance genes such as KPC, OXA-48 and mcr-1 in the environment.

We used conventional PCR to identify carbapenemase resistance genes; KPC genes were detected in fly-borne *E. coli*, *Enterobacter aerogenes* and *Proteus mirabilis*. These genes were first identified in Latin America in Colombia in 2004 ^20^ and in Peru in 2013 ^21^. OXA-48 genes were found in *Klebsiella oxytoca*, *E. coli*, *Enterobacter cloacae*, and *Proteus vulgaris*. These genes are prevalent in *Klebsiella pneumoniae* and other enterobacteria and have also been detected in *Acinetobacter baumannii*. In Peru in 2022, seven strains of bacteria carrying the OXA-48 gene were isolated from patients at the National Institute of Neoplastic Diseases ^22^.

In our study, a strain of *E. coli* with the mcr-1 gene that confers resistance to colistin was detected, a result similar to findings in China ^8^, India ^23^^)^ and Peru ^24^. Bacteria carrying carbapenemase-encoding genes and carrying the mcr-1 gene in our study showed resistance to most antibiotics, except carbapenems and gentamicin.

There are no previous studies on the identification of antibiotic resistance genes in bacteria isolated from flies in Peru, which prevents comparative analysis. In addition, the exclusive use of the mcr-1 primer could have limited the detection of other allelic variants, and the restricted availability of positive controls could have hindered the use of primers to identify these additional allelic variants (mcr-2 to mcr-10) in the Peruvian context. The number of flies captured in this study could be considered a limitation to obtain more solid conclusions; however, since this is the first report of antibiotic resistance genes in bacteria isolated from *Musca domestica* in the country, it will serve as a starting point for future research.

In conclusion, our results show that *Musca domestica* can be considered as a potential disseminator of bacteria carrying antibiotic resistance genes, specifically genes for carbapenemases and mcr-1 for plasmid resistance to colistin. We recommend that more complex studies should be carried out to identify a greater number of resistance genes to different antibiotics, considering more than 300 flies per sampling area, as applied in the study carried out in China and India, and thus be able to evaluate with greater certainty the role of the house fly as a disseminator of bacteria with antibiotic resistance and its possible implications for public health.
