# Supplementary material for: Carbapenems and colistin resistance genes isolated in Musca domestica from a garbage dump near a hospital in Lima
Source: Rev Peru Med Exp Salud Publica. 2024 Jun 12;41(2):164–70. doi: 10.17843/rpmesp.2024.412.13257 (PMC11300682; doi:10.17843/rpmesp.2024.412.13257)
Supplement: Translation

# Translation: Genes resistentes a carbapenémicos y colistina aislados en *Musca domestica* proveniente de un basural cercano a un hospital de Lima

**Authors:** Miguel A. Alarcón-Calle, Víctor L. Osorio-Guevara, Ramsés Salas-Asencios, José Yareta, Pool Marcos-Carbajal, María E. Rodrigo-Rojas

**Affiliations:** 1 Laboratorio de Investigación en Biología, Facultad de Ciencias Naturales y Matemática, Universidad Nacional Federico Villarreal, Lima, Perú.; 2 Laboratorio de Biotecnología, Facultad de Ciencias Naturales y Matemática, Universidad Nacional Federico Villarreal, Lima, Perú.; 3 Laboratorio de Investigación en Biología Molecular, Escuela Profesional de Medicina, Universidad Peruana Unión, Lima, Perú.; 4 Laboratorio de Epidemiología Molecular y Genómica, Instituto de Investigación en Ciencias Biomédicas, Facultad de Medicina Humana, Universidad Ricardo Palma, Lima, Perú.

**Keywords:** Moscas Domésticas, Farmacorresistencia Microbiana, Colistina, Carbapenémicos

## Abstract

El objetivo fue determinar la presencia de genes de resistencia a carbapenémicos y resistencia plasmídica a colistina (mcr-1) en bacterias aisladas de *Musca domestica* en un basural cercano a un hospital de Lima, Perú. Las bacterias con resistencia fenotípica a los carbapénemicos se aislaron en medio CHROMagar mSuperCARBA^TM^ y el perfil de resistencia a colistina se realizó mediante el método de elución de discos de colistina. La detección de genes *bla*KPC, *bla*NDM, *bla*IMP, *bla*OXA-48, *bla*VIM y mcr-1 se realizó mediante PCR convencional. El perfil de susceptibilidad antimicrobiana se determinó mediante el sistema automatizado MicroScan. Las bacterias con resistencia fenotípica a carbapenémicos fueron 31/38 cepas y a colistina fueron 26/38 cepas con una concentración inhibitoria mínima ≥ 4 µg/ml. Finalmente, se identificaron siete cepas bacterianas con genes de resistencia a carbapenémicos (OXA-48 Y KPC) y una cepa bacteriana con resistencia plasmídica a colistina (mcr-1). Una cepa de *Escherichia coli* presentó tres genes de resistencia: KPC, OXA-48 y mcr-1.

## INTRODUCCIÓN

La Organización Mundial de la Salud (OMS) alerta sobre la resistencia antimicrobiana (RAM) a nivel global, con el aumento de bacterias resistentes a múltiples fármacos (MDR) y la disminución de opciones terapéuticas. Esto causa aproximadamente 700 000 muertes anuales, estimando 10 millones de muertes anuales para el 2050, afectando factores socioeconómicos y demográficos ^(^^1^.

Los carbapenémicos han sido considerados como «el último y el más efectivo recurso para tratar las infecciones bacterianas» ^2^; pero, se ha observado un incremento de bacterias productoras de carbapenemasas, como *Klebsiella pneumoniae, Acinetobacter baumannii y Pseudomonas aeruginosa*^3^. Diversas enzimas, como Verona metalo-beta-lactamasa (VIM), *Klebsiella pneumoniae* carbapenemasa (KPC), Imipenemasa (IMP), Oxacilinasas (OXA-48), y la New Delhi metalo-betalactamasa (NDM), se han descrito desde la década de 1990 con diseminación global, incluido Perú ^4^. El uso de la colistina como último recurso terapéutico se ha visto obstaculizado por la aparición del gen de resistencia Mobile Colistin Resistance (mcr-1) en China en el 2015, propagándose mediante diferentes orígenes ^5^. El primer reporte en Perú relacionado con el gen mcr-1 fue realizado en *Escherichia coli* (*E. coli)* proveniente de una muestra de urocultivo ^6^.

Las moscas domésticas son vectores mecánicos y biológicos de bacterias, portando hasta 500 000 agentes bacterianos, incluyendo patógenos clínicos ^7^. Zhang *et al*. detectaron genes de resistencia a colistina (mcr-1 a mcr-3) en moscas, con el 34,1% de bacterias positivas a mcr-1 ^8^.

En Brasil se reportó por primera vez en *Musca domestica* el gen *bla*NDM-1 en *E. coli* proveniente de un centro urbano en Río de Janeiro ^9^. Sin embargo, existen pocos reportes respecto a bacterias productoras de carbapenemasas y resistentes a colistina transportadas por la *Musca domestica*. No existen estudios en Perú sobre vectores como fuente de diseminación de genes de resistencia a este grupo de antibióticos. Este estudio busca identificar genes productores de carbapenemasas y gen plasmídico mcr-1 en bacterias aisladas a partir de moscas de un basural cercano a un hospital en Lima, en Perú.

**MENSAJES CLAVE**

Motivación para realizar el estudio. La presencia de genes de resistencia a antibióticos en bacterias aisladas de moscas comunes es un peligro potencial para la salud pública debido a que facilita la presencia y dispersión de genes de resistencia a antibióticos en el medio ambiente.

Principales hallazgos. Se aislaron 38 cepas bacterianas identificadas en 14 especies dentro del cuerpo de las moscas, de las cuales 31 cepas mostraron resistencia a los carbapenémicos y 26 cepas mostraron resistencia a colistina. Siete cepas bacterianas presentaron genes de resistencia a carbapenémicos y una cepa de *Escherichia coli* con resistencia a KPC, OXA-48 y mcr-1.

Implicancias. Se realiza el primer reporte en el Perú de genes de resistencia a antibióticos en bacterias movilizadas por moscas comunes.

## EL ESTUDIO

Los ensayos del estudio se realizaron en los Laboratorios de Investigación en Biología de la Universidad Nacional Federico Villarreal y el Laboratorio de Investigación en Biología Molecular (LIBM) de la Universidad Peruana Unión durante marzo a agosto del 2023. 

### Diseño y muestra

Se llevó a cabo un estudio observacional, transversal y descriptivo en el cual se analizaron 30 moscas domésticas recolectadas en abril del 2023 en un basural cercano al Hospital Nivel II de EsSalud en Ate Vitarte, Lima, Perú (coordenadas: -12.026535/-76.924063).

### Colecta e identificación de la mosca doméstica

Para la colecta e identificación de las moscas, se utilizaron platos adhesivos con cebo de pollo como carnada. Cada mosca fue colocada individualmente en tubos Eppendorf de 2 ml, manteniéndola a 0 °C durante 20 minutos para adormecerla y facilitar su manipulación ^9^. Luego, se procedió a su identificación utilizando la clave taxonómica de Robert Moon ^10^.

### Cultivo, identificación bacteriana y perfil de susceptibilidad

Se sometieron las moscas a un proceso de lavado con PBS 1X estéril para eliminar bacterias presentes en las moscas por contacto externo. Luego se trituraron las moscas y el homogenizado se resuspendió en PBS para ser centrifugado durante 10 minutos. Se extrajeron 1000 µl del sobrenadante y se colocaron en tubos con 3 ml de caldo tripticasa de soya y se incubó a 37 °C entre 18 a 24 horas ^8^; posteriormente se extrajo 10 µl de caldo con una asa calibrada y se sembró en placas con agar MacConkey. La identificación bacteriana se realizó mediante pruebas bioquímicas convencionales: Agar TSI, LIA, SIM, Citrato y Úrea.

El perfil de susceptibilidad a los antibióticos y la confirmación de especies, para las cepas con genes de resistencia determinadas por PCR, se realizó con el sistema automatizado Microscan® (AutoScan-4) siguiendo las instrucciones de manufactura. En el panel de susceptibilidad se incluyeron 18 antimicrobianos: amikacina (AK), gentamicina (GEN), tobramicina (TOB), cefepime (FEP), cefuroxima (CFX), ceftazidima (CAZ), cefotaxima (FOX), ampicilina (AMP), ampicilina con sulbactam (AMP/SUL), amoxicilina con ácido clavulánico (AMC), piperacilina con tazobactam (PIP/TZ), imipenem (IMI), meropenem (MEM), ertapenem (ETP), aztreonam (ATM), ciprofloxacina (CIP), levofloxacina (LEV), trimetoprim/sulfametoxazol (SXT) y colistina (COL). Los resultados fueron interpretados según las pautas del Clinical and Laboratory Standards Institute (CLSI, por sus siglas en inglés) ^11^.

### Detección fenotípica de carbapenemasas y resistencia a colistina

El aislamiento de bacterias con resistencia fenotípica a los carbapenémicos se realizó en el medio CHROMagar^TM^ mSuperCARBA^TM^. Para evaluar la resistencia fenotípica a colistina, se emplearon discos de colistina de 10 µg en caldo Müller Hinton con cationes, siguiendo las pautas del Instituto de Estándares Clínicos y de Laboratorio (CLSI) 2023, considerando resistencia con un punto de corte ≥ 4 µg/ml ^11^.

### Detección de genes *bla*KPC, *bla*NDM, *bla*IMP, *bla*OXA-48, *bla*VIM y mcr-1

Finalmente, se extrajo el ADN utilizando el kit inmuPREP Bacteria DNA Kit (Analitikjena, Alemania) y se detectaron genes de resistencia *bla*KPC ^12^, *bla*NDM ^13^, *bla*IMP ^14^, *bla*OXA-48 ^15^, *bla*VIM ^16^ y mcr-1 ^17^ mediante Reacción en Cadena de la Polimerasa en un termociclador T100™ (Biorad, USA), siguiendo protocolos estandarizados en el laboratorio (LIBM); en el caso del gen mcr-1 se utilizó el protocolo de Iglesias ^17^. Los cebadores utilizados para este proceso se detallan en la tabla 1. Se utilizaron cepas bacterianas multidrogorresistentes como controles positivos, tales como *Klebsiella pneumoniae* derivado de ATCC BAA-1705 (Microbiologics) para el gen KPC, *Escherichia coli* derivado de ATCC BAA-2469 (Microbiologics) para el gen NDM. Asimismo, cepas proveídas por el Laboratorio de Referencia Nacional (Laboratorio Intrahospitalaria-LIH) del Instituto Nacional de Salud para cada gen de interés (IMP, OXA-48, VIM y mcr-1) identificados mediante PCR; y dos controles negativos: agua grado molecular y *Escherichia coli* ATCC 25922 (Microbiologics).

**Tabla 1:** Cebadores utilizados en el presente estudio.

Gen	Cebado	Secuencia 5’-3’	Amplicón	Referencia
KPC	F	AACAAGGAATATCGTTGATG	916 pb	^12^
	R	AGATGATTTTCAGAGCCCTTA		
NDM	F	AGCACACTTCCTATCTCGAC	512 pb	^13^
	R	GGCGTAGTGCTCAGTGTC		
IMP	F	GGYGTTTWTGTTCATACWTCKTTYGA	404 pb	^14^
	R	GGYARCCAAACCACTASGTTATCT		
VIM	F	AGTGGTGAGTATCCGACAG	261 pb	^15^
	R	ATGAAAGTGCGTGGAGAC		
OXA-48	F	ATGCGTGTATTAGCCTTATCGG	438 pb	^16^
	R	GCGTGGTTAAGGATGAACAC		
MCR-1	F	CGGTCAGTCCGTTTGTTC	309 pb	^5^
	R	CTTGGTCGGTCTGTAGGG		

F: Forward, R: Reverse, KPC: *Klebsiella pneumoniae* Carbapenemase, NDM: New Delhi metallo-β-lactamase, IMP: Imipenemasa, VIM: Verona metalo-beta-lactamasa codificada por integrón, OXA 48: Oxacilinasa tipo 48 y MCR: Mobile Colistin Resistance

### Electroforesis

Los productos de amplificación se analizaron mediante electroforesis en gel de agarosa al 1,5% (Cleaver Scientific) con SYBR Safe DNA Gel Stain como tampón de gel, y 6X DNA Loading Dye como tampón de carga. Se empleó un marcador de 100 pb DNA(Trans) para evaluar el tamaño del ADN, con una corrida a 100 V durante 40 minutos.

### Análisis estadístico

Se utilizó el software IBM SPSS Statistics 25.0 para presentar las medidas de frecuencias en forma de porcentajes. Las tablas y figuras se realizaron mediante Microsoft Excel 2019.

### Consideraciones éticas

Dado que no se manejaron datos de pacientes, no se requirió aprobación de un comité institucional de ética.

## HALLAZGOS

Se aislaron 38 cepas bacterianas de las cuales se identificaron 14 especies, tal como se detalla en la tabla 2. *Klebsiella oxytoca* fue la más prevalente (10/38, 23,7%) seguida de *Escherichia coli* (7/38, 21,1%).

**Tabla 2:** Frecuencia de bacterias aisladas, detección fenotípica y genotípica relacionadas con la producción de carbapenemasas y resistencia plasmídica a colistina.

Bacterias	Fenotipo			Genotipo			
	Frecuencia (%)	mSuperCARBA ^TM^ (%)	Elución de discos de colistina ≥4µg/ml (%)	Gen			
				***bla*KPC**	***bla*NDM**	***bla*OXA-48**	***bla*VIM**
*Klebsiella oxytoca*	10 (23,7)	10 (32,3)	5 (19,2)	ND	ND	1	ND
*Escherichia coli*	7 (21,1)	7 (22,6)	5 (19,2)	1	ND	2	ND
*Enterobacter aerogenes*	3 (7,9)	2 (6,5)	3 (11,5)	1	ND	ND	ND
*Citrobacter freundii*	3 (7,9)	2 (6,5)	2 (7,7)	ND	ND	ND	ND
*Enterobacter cloacae*	2 (5,3)	2 (6,5)	2 (7,7)	ND	ND	1	ND
*Klebsiella pneumoniae*	2 (5,3)	2 (6,5)	1 (3,8)	ND	ND	ND	ND
*Proteus vulgaris*	2 (5,3)	1 (3,2)	1 (3,8)	ND	ND	1	ND
*Pseudomonas aeruginosa*	2 (5,3)	1 (3,2)	1 (3,8)	ND	ND	ND	ND
*Pseudomonas fluorescens*	2 (5,3)	1 (3,2)	1 (3,8)	ND	ND	ND	ND
*Acinetobacter baumannii*	1 (2,6)	0 (0,0)	1 (3,8)	ND	ND	ND	ND
*Morganella morganni*	1 (2,6)	1 (3,2)	1 (3,8)	ND	ND	ND	ND
*Proteus mirabilis*	1 (2,6)	1 (3,2)	1 (3,8)	1	ND	ND	ND
*Proteus penneri*	1 (2,6)	0 (0,0)	1 (3,8)	ND	ND	ND	ND
*Serratia marcescens*	1 (2,6)	1 (3,2)	1 (3,8)	ND	ND	ND	ND
Total	38 (100,0)	31 (100,0)	26(100,0)	3	0	5	0

ND: No detectado, KPC: *Klebsiella pneumoniae* Carbapenemase, NDM: New Delhi metallo-β-lactamase, IMP: Imipenemasa, VIM: Verona metalo-beta-lactamasa codificada por integrón, OXA 48: Oxacilinasa tipo 48 y MCR: Mobile Colistin Resistance

Se aislaron 31 cepas de bacterias con resistencia fenotípica a carbapenémicos: *Klebsiella oxytoca* (10/31, 32,3%) y Escherichia coli (7/31, 22,6%) fueron las de mayor frecuencia. Además, 26 cepas presentaron resistencia fenotípica a colistina; donde *Escherichia coli* y *Klebsiella oxytoca* mostraron la mayor resistencia (5/26, 19,2%), todas con punto de corte ≥4µg/ml.

Se detectaron siete cepas bacterianas con genes de resistencia a carbapenémicos (OXA-48 y KPC) aisladas en CHROMagar^TM^ mSuperCARBA^TM^, de las cuales se encontró el gen *bla*KPC en *Enterobacter aerogenes* (1/2) y *Proteus mirabilis* (1/1) y el gen *bla*OXA-48 en *Escherichia coli* (1/7), *Klebsiella oxytoca* (1/10), *Enterobacter cloacae* (1/2) y *Proteus vulgaris* (1/2). Además, se detectó una cepa de *Escherichia coli* (1/7) con los genes de resistencia *bla*KPC, *bla*OXA-48 y al gen plasmídico mcr-1 asociado a colistina, que representaría el 3,8 % de 1/26 cepas. Detalles del gen mcr-1 en la figura 1 (corrida electroforética) y figura 2 (mapa de calor, perfil de resistencia fenotípica y genotípica).

**Figura 1:** Resultados de la electroforesis para la detección del gen mcr-1.

En cuanto al perfil de resistencia a los antibióticos, todos los aislados que presentaron genes de resistencia detectados fueron resistentes a 1 o 2 grupos de antibióticos, tal como se detalla en la figura 2. Por otro lado, la resistencia bacteriana a trimetoprima/sulfametoxazol fue del 100%, a ampicilina 85,7% y a ciprofloxacina 42,9%, mientras que hubo sensibilidad y/o resistencia intermedia para los antibióticos ertapenem, imipenem y meropenem en las siete cepas. También se observó resistencia a colistina en las cepas de *Proteus*; sin embargo, esta resistencia es natural de la bacteria.

**Figura 2:** Perfil de resistencia genotípica y fenotípica de bacterias productoras de carbapenemasas.

## DISCUSIÓN

Los resultados obtenidos en el presente trabajo muestran que las moscas no sólo sirven como vectores de dispersión de diferentes especies de bacterias, sino también de bacterias portadoras de genes de resistencia a carbapenémicos y a colistina (mcr-1).

En el 2021 se realizó un estudio en Sudán, África, con 300 moscas colectadas en los mataderos y hospitales, examinando bacterias externas e internas de cada mosca, se identificaron 283 bacterias en moscas de hospitales y 366 bacterias en moscas de mataderos; siendo *E. coli*, *la bacteria con mayor frecuencia de aislamiento*^18^. A pesar de la diferencia en el tamaño de la muestra bacteriana aislada en nuestro estudio, se detectaron un 32,2% de *Klebsiella oxytoca* y un 22,6% de *E. coli,* todas productoras de carbapenemasas; siendo por tanto las especies de bacterias transportadas con mayor frecuencia por *Musca domestica*.

En el 2004 y 2005, en mercados y basurales de Lima y Callao (Perú), se recolectaron 780 moscas, y se identificaron *Salmonella typhi, Shigella flexneri, Yersinia enterocolitica* y *E. coli* enteropatógena ^19^. Estos resultados permitieron a los autores señalar la importancia de las moscas como vectores mecánicos para la dispersión de bacterias en el medioambiente; nuestros resultados también muestran a *E. coli* como una bacteria aislada de *Musca domestica* que habitan los basurales y que además podría dispersar genes de resistencia tales como: KPC, OXA-48 y mcr-1 a través del medioambiente.

En nuestro estudio, utilizamos PCR convencional para identificar genes de resistencia a carbapenemasas; se detectaron genes KPC en *E. coli, Enterobacter aerogenes* y *Proteus mirabilis* transmitidas por moscas. Estos genes se identificaron por primera vez en Latinoamérica en Colombia en 2004 ^20^ y en Perú en 2013 ^21^. Genes OXA-48 fueron encontrados en *Klebsiella oxytoca, E. coli, Enterobacter cloacae* y *Proteus vulgaris*. Estos genes prevalecen en Klebsiella pneumoniae y otras enterobacterias y también se han detectado en Acinetobacter baumannii. En Perú el 2022 se aislaron siete cepas de bacterias portadoras del gen OXA-48 en pacientes del Instituto Nacional de Enfermedades Neoplásicas ^22^.

En el presente estudio se detectó una cepa de *E. coli* con el gen mcr-1 que confiere resistencia a colistina, resultado similar a hallazgos en China ^8^, India ^23^ y Perú ^24^. Las bacterias portadoras de genes que codifican carbapenemasas y portadoras del gen mcr-1 en nuestro estudio mostraron resistencia a la mayoría de los antibióticos, excepto carbapenémicos y gentamicina.

En Perú, no se han llevado a cabo estudios previos sobre la identificación de genes de resistencia a antibióticos en bacterias aisladas de moscas, lo que ha impedido la realización de un análisis comparativo. Además, la exclusiva utilización del cebador mcr-1 pudo haber limitado la detección de otras variantes alélicas, y la disponibilidad restringida de controles positivos podría haber dificultado el uso de cebadores para identificar estas variantes alélicas adicionales (mcr-2 al mcr-10) en el contexto peruano. La cantidad de moscas capturadas en este estudio podría considerarse una limitación para obtener conclusiones más sólidas; sin embargo, dado que este es el primer informe de genes de resistencia a antibióticos en bacterias aisladas de Musca doméstica en el país, servirá como punto de partida para investigaciones futuras.

En conclusión, los resultados obtenidos muestran que se puede considerar a *Musca domestica* como potencial diseminador de bacterias portadoras de genes de resistencia a antibióticos, específicamente genes para carbapenemasas y mcr-1 para resistencia plasmídica a colistina. Es recomendable que se realicen estudios de mayor complejidad que permitan identificar un mayor número de genes de resistencia a diferentes antibióticos, considerando más de 300 moscas por zona de muestreo, como se aplicó en los trabajos realizados en China e India; y así poder evaluar con mayor certeza el rol que cumple la mosca doméstica como agente diseminador de bacterias con resistencia a antibióticos y sus posibles implicancias sobre la salud pública.
